# Microscopic dynamics of synchronization in driven colloids

**DOI:** 10.1038/ncomms8187

**Published:** 2015-05-21

**Authors:** Michael P.N. Juniper, Arthur V. Straube, Rut Besseling, Dirk G.A.L. Aarts, Roel P.A. Dullens

**Affiliations:** 1 Department of Chemistry, Physical and Theoretical Chemistry Laboratory, University of Oxford, South Parks Road, Oxford OX1 3QZ, UK; 2 Department of Physics, Humboldt-Universität zu Berlin, Newtonstraße 15, Berlin 12489, Germany; 3 InProcess-LSP, Molenstraat 110, Oss 5342 CC, Netherlands

## Abstract

Synchronization of coupled oscillators has been scrutinized for over three centuries, from Huygens' pendulum clocks to physiological rhythms. One such synchronization phenomenon, dynamic mode locking, occurs when naturally oscillating processes are driven by an externally imposed modulation. Typically only averaged or integrated properties are accessible, leaving underlying mechanisms unseen. Here, we visualize the microscopic dynamics underlying mode locking in a colloidal model system, by using particle trajectories to produce phase portraits. Furthermore, we use this approach to examine the enhancement of mode locking in a flexible chain of magnetically coupled particles, which we ascribe to breathing modes caused by mode-locked density waves. Finally, we demonstrate that an emergent density wave in a static colloidal chain mode locks as a quasi-particle, with microscopic dynamics analogous to those seen for a single particle. Our results indicate that understanding the intricate link between emergent behaviour and microscopic dynamics is key to controlling synchronization.

## Abstract (web summary)

Synchronization may occur when naturally oscillating systems are driven by an external modulation, for example, in charge density waves. Here, Juniper *et al*. visualize the locked modes of synchronization at a microscopic level using a colloidal system.

## Graphical abstract

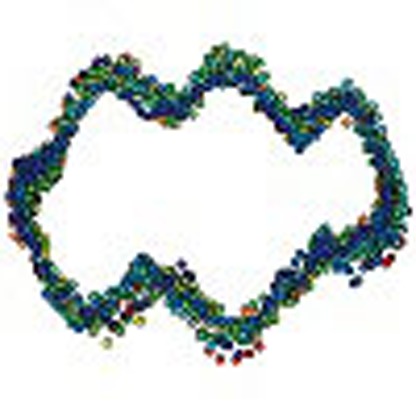

In the natural world, little exists in a state of true equilibrium; in fact, most of the physics around us is far from equilibrium^1^. On both macroscopic and microscopic levels, driven systems exhibit rich and complex behaviour well beyond their equilibrium properties, due to the delicate balance between the external drive and the ‘internal' equilibrium behaviour opposing the drive^2^,^3^,^4^,^5^,^6^,^7^,^8^,^9^,^10^.

Driving a system with a natural internal oscillation by an external modulation leads to a synchronization phenomenon termed dynamic mode locking^11^. Coupled oscillators have been studied since Huygens' observation of synchronization between pendulum clocks in 1665 (refs 11, 12, 13, 14, 15). In the case of dynamic mode locking, coupling between the ‘internal' and ‘external' frequencies leads to synchronization into repeating modes of motion^11^. Much effort has gone into the understanding of this technologically important effect in vortex lattices^16^,^17^,^18^,^19^,^20^, laser gyroscopes^21^, charge density waves^22^,^23^,^24^ and Josephson junctions^25^,^26^,^27^. In all of these cases, however, typically only averaged or integrated properties are probed, leaving a direct visualization of the underlying dynamics lacking.

The use of experimental model systems composed of colloidal particles in external fields is well established, due to their inherently accessible time and length scales, and ease of manipulation^28^,^29^. From stochastic resonance in double-well potentials^30^,^31^,^32^ to directed motion on optical lattices^7^,^33^, study of such systems has proved fruitful. Despite extensive theoretical treatment^34^,^35^,^36^,^37^,^38^ and simulations^39^,^40^,^41^,^42^,^43^, the difficulty of incorporating the complex hydrodynamics means that experimental approaches continue to be valuable^44^,^45^,^46^,^47^, also as a benchmark for theoretical and simulation studies.

In this article, we reveal the microscopic dynamics underlying dynamic mode locking by driving colloidal particles across a periodic potential energy landscape and observing them in real space and time. Having access to particle trajectories allows us to visualize the nature of the oscillating particle motion, and differentiate locked from unlocked states. Moreover, we are able to distinguish between modes which have the same average velocity, which are indistinguishable by studying only integrated properties^17^,^18^,^22^,^25^,^48^,^49^. The microscopic approach is also illuminating when applied to more complex collective behaviour. We study a colloidal chain with tunable flexibility, and find that mode locking is enhanced when the chain length is allowed to vary. Length fluctuations are found to be periodic breathing modes, which are tied to the velocity oscillations of the chain as a whole. Breathing modes in finite driven chains may be caused by density waves^50^, manifesting as caterpillar-like motion. We therefore engineer a density wave or ‘kink' in a pinned colloidal chain, and demonstrate that it behaves as a quasi-particle, mode locking in a manner analogous to single driven particles. By showing that a density wave displays dynamic mode locking and classifying its phase portraits, we demonstrate the wider applicability of our approach to studying synchronization in complex driven systems^6^,^7^,^20^,^38^,^41^.

## Results

### Colloidal dynamic mode locking

**Figure 1:**
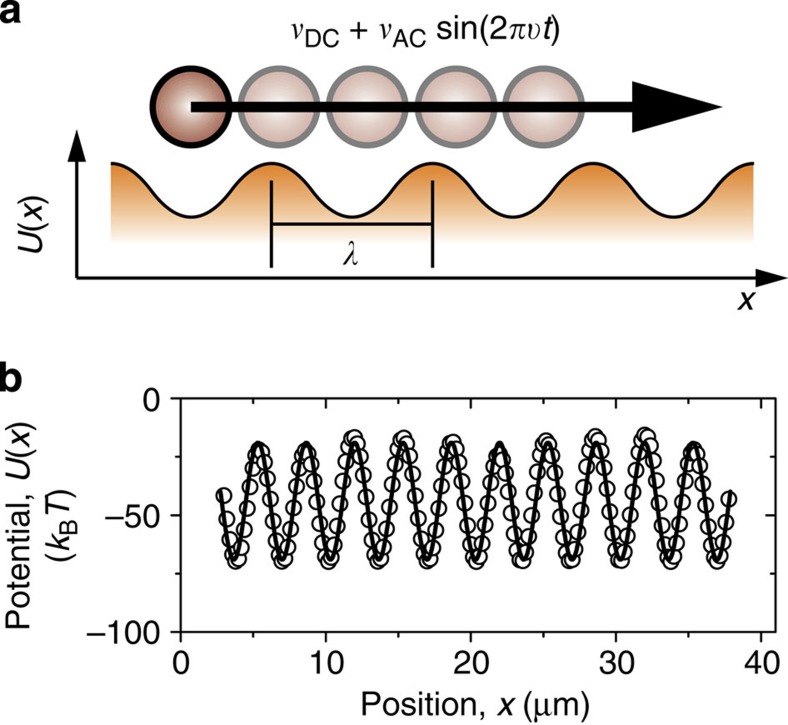
Driving particles across a sinusoidal potential energy landscape. (**a**) The particles are driven with a constant, *v*_DC_, and a modulated (AC) driving velocity with amplitude *v*_AC_ and frequency *ν*. *λ* is the wavelength of the landscape. (**b**) Measurement of part of a typical optical potential energy landscape, as described in ref. 51, fitted with a sine function. Trap spacing: *λ*=3.5 μm; laser power per trap *W*≈0.75 mW.

**Figure 2:**
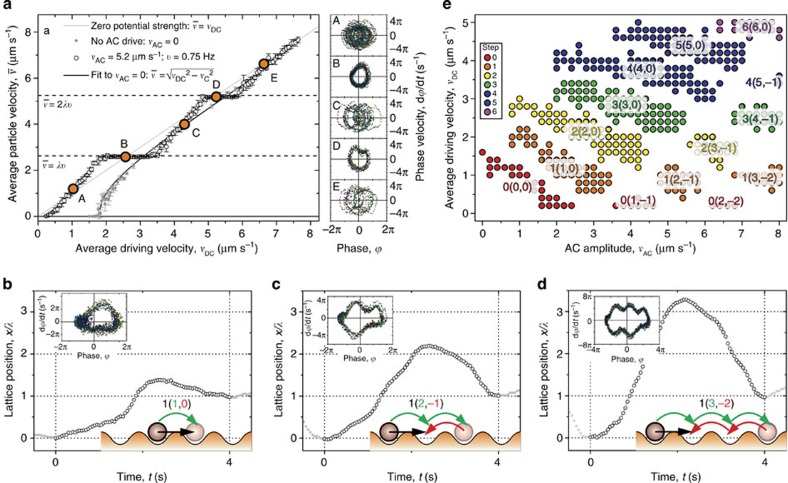
Microscopic dynamics of dynamic mode locking. A particle is driven over a sinusoidal optical potential energy landscape with wavelength *λ*=3.5 μm. (**a**) Average particle velocity 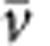 as a function of average driving velocity *v*_DC_. Solid grey points: zero AC drive. Open circles: AC drive with amplitude *v*_AC_=5.2 μm s^−1^ and frequency *ν*=0.75 Hz. Solid grey line shows 
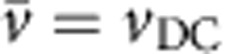
 for the case of zero potential strength. All points are the result of six repeats, error bars show the standard deviation of repeats. Phase portraits A–E correspond to the highlighted points on the graph. (**b**–**d**) Single period trajectories and phase portraits corresponding to 1(1,0), 1(2,−1) and 1(3,−2) modes for particles driven with AC frequency *ν*=0.25 Hz. (**e**) State diagram showing the dynamic modes over a range of AC and DC drives, for *ν*=0.25 Hz. Coloured regions are locked states. *n*(*i*,*j*) indicates the net forward motion *n*, and the number of lattice spacings moved forward (*i*) and backward (*j*). Each point is confirmed in two repeats.

Polystyrene particles with a diameter of 3 μm, dispersed in a water–ethanol mixture, are driven using a piezo stage across a one-dimensional potential energy landscape with wavelength *λ* generated with optical tweezers^51^, as shown in Fig. 1a. The landscape is designed such that there is a significant barrier to diffusive particle motion between its minima, but also so that this barrier may be overcome by modest driving velocities. The landscape must be periodic, so a sinusoidal form is generated by placing the optical traps sufficiently close to each other^51^. Figure 1b shows a measurement of such a potential landscape with a wavelength of *λ*=3.5 μm and a laser power per trap of *W*≈0.75 mW (trap stiffness: *k*=3.8 × 10^−7^ kg s^−2^; trap depth: *V*_0_=75 *k*_B_*T*), measured using a driven colloidal particle as described in ref. 51. The particle may be driven by both a constant (DC) velocity, *v*_DC_, and a modulated (AC) velocity, *v*_AC_ sin(2π*νt*), with *ν* the driving frequency (see Fig. 1a). First, in Fig. 2a (grey points), we show the mean velocity, 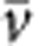, of a particle driven with a constant driving velocity only (*v*_AC_=0). The mean particle velocity sharply increases from zero when the driving velocity exceeds the critical velocity and then follows the well-known relation 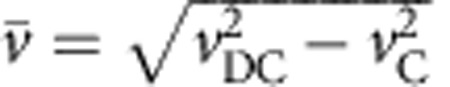, where *v*_C_ is the critical velocity below which a particle is pinned by the landscape^52^.

To induce dynamic mode locking, the constant driving velocity *v*_DC_ is supplemented by a modulated driving velocity, *v*_AC_ sin (2π*νt*). Adding a modulated drive leads to completely different dynamics, as shown by the open circles in Fig. 2a. Now the average particle velocity increases non-uniformly, with ‘steps' where 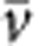 remains constant over a range of *v*_DC_, indicating synchronization. These plateaus are known as ‘Shapiro steps' as seen in Josephson junctions^25^,^26^,^48^,^49^ and vortex lattices^17^,^18^,^19^. The average velocity at the dynamic mode locking steps is given by the resonance condition 
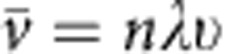
, where *n* is an integer step number. The ratio 
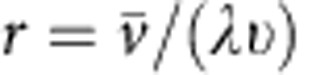
 is the rotation number, which for a locked state in our system is always strictly integer. It is interesting to note that this resonance condition is independent of the particle size, even though the critical driving velocity is not.

### Phase portraits

From the particle trajectories, we can directly visualize the periodic motion at resonance. We construct so-called ‘phase portraits', in which the phase velocity, d*ϕ*/d*t*, is plotted against the phase, 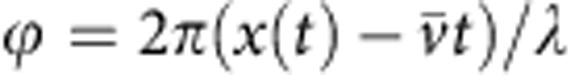 (ref. 53). The phase portrait shows a ‘closed loop' if the particle is in a locked state and thus follows a perfectly periodic trajectory with an integer rotation number. Otherwise, the phase portrait shows an ‘open loop', as each cycle is slightly different and the phase trajectory is quasi-periodic with a non-integer rotation number. The phase portraits corresponding to different average velocities (points A–E) are presented in Fig. 2a. Those for A, C and E indeed show open loops as they lie between steps where there is no synchronization. In contrast, the points on the resonant steps (B and D, with *r*=1,2) clearly show closed loops in their phase portraits.

The phase portraits also strikingly depict the microscopic nature of the modes. Importantly, we can visualize differences between modes with the same average velocity, which is not possible when only considering integrated quantities. We illustrate this in Fig. 2b–d, by showing three trajectories of mode-locked particles with the same average velocity over one period of the oscillation. In Fig. 2b, the particle simply moves forward by one lattice spacing in each period. In Fig. 2c, the net motion is the same, but the particle moves forward two and back one lattice spacing, and in Fig. 2d, it moves forward three and backward two, as indicated in the schematics. So, it is clear that the microscopic particle dynamics differ significantly for each mode, even though they all have the same average velocity. We label the modes *n*(*i*,*j*), with *n* the net forward motion, and *i* and *j* the number of lattice spacings moved forward and backward, respectively, so that *n*=*i*+*j*. The nature of the modes is also evident from the phase portraits, where the number of ‘bumps' in the top and bottom halves correspond to *i* and *j*.

**Figure 3:**
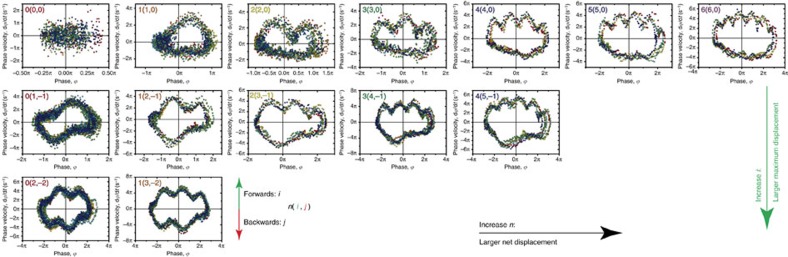
Phase portraits for all of the modes shown in Fig. 2e. The modes are labelled as *n*(*i*,*j*), with *n* the net forward motion, and *i* and *j* the number of lattice spacings moved forward and backward respectively, such that *n*=*i*+*j*.

All of the dynamic modes in a portion of *v*_DC_–*v*_AC_ space are displayed on the state diagram in Fig. 2e and the complex distribution of the modes highlights the rich dynamics. A vertical cut through the state diagram corresponds to a ‘staircase' as shown in Fig. 2a and the colour corresponds to the step number *n* in the resonance condition 
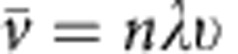
. Subsequently, each colour can be split into several regions, corresponding to different modes with distinct particle motion as characterized by *n*(*i*,*j*). The corresponding phase portraits are shown in Fig. 3. The oscillating size of the regions with the same average velocity corresponds to twisted ‘Arnold Tongues', seen in many synchronized systems^11^. For high average velocities, where the potential can be accounted for perturbatively, the oscillating step width is well described by a Bessel function^19^.

Although Arnold Tongues are continuous, in this noisy Brownian system, gaps between the modes appear on the state diagram. This means that at some values of the AC amplitude, steps will not appear for all velocities satisfying the resonance condition. For example, at *v*_AC_=6.4 μm s^−1^, there are no steps corresponding to *n*=1 or *n*=3, even though steps exist for *n*=0,2,4,5 and 6. It is also worth noting that regions occur where there is no *n*=0 step, which means that there is no effective critical driving velocity, and that the particle will always have net forward motion as long as *v*_DC_>0. The *n*=0 modes are particularly interesting, as they demonstrate that a lack of net motion does not necessarily imply that the system is at rest. This is another insight that would be absent from only studying the integrated properties of mode-locked steps.

### Driven colloidal chains

**Figure 4:**
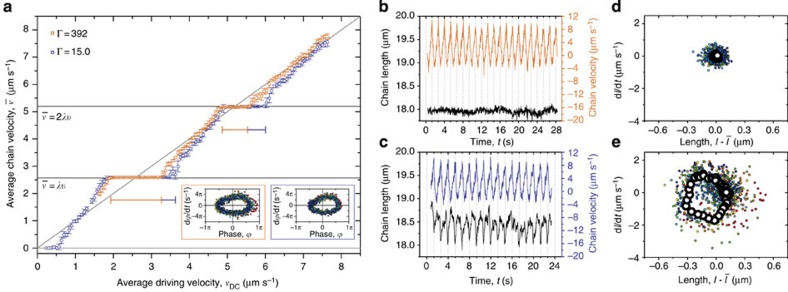
Dynamic mode locking in driven chains. Linear chains of seven magnetically coupled particles are driven over a sinusoidal optical potential energy landscape with wavelength *λ*=3.5 μm, by a DC and AC drive with amplitude *v*_AC_=5.2 μm s^−1^ and frequency *ν*=0.75 Hz. (**a**) Average chain velocity 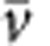 as a function of average driving velocity *v*_DC_. Purple squares: dipolar interaction strength Γ=15; orange circles: Γ=392. Solid line shows 
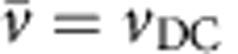
 for the case of zero potential strength. All points are the result of six repeats, error bars show the standard deviation of repeats. Insets show the phase portraits for the chain position for the first step. (**b**,**c**) The chain length and velocity for the first step, for the strongly and weakly coupled chains, respectively. (**d**,**e**) Phase portraits for the chain length for the strongly and weakly coupled chains, respectively. Open circles show average phase trajectories.

We now consider the dynamics of driven coupled systems as dynamic mode locking is often found as a collective effect^5^,^18^,^19^,^54^,^55^. We drive a chain consisting of seven super-paramagnetic colloidal particles held together by attractive magnetic interactions over the same sinusoidal optical potential energy landscape used above (trap spacing: *λ*=3.5 μm; laser power per trap: *W*≈0.75 mW; trap stiffness: *k*=3.8 × 10^−7^ kg s^−2^; trap depth: *V*_0_=75 *k*_B_*T*). We tune the flexibility of the chain via the interaction parameter, Γ, which is the strength of the magnetic interaction between a pair of particles at contact in units of *k*_B_*T* (see Methods). A magnetic field is applied using a pair of identical permanent magnets positioned equidistant from the centre of the sample, and the magnetic field strength *B* (and therefore Γ, as Γ∝*B*^2^) is varied by altering this distance. Chain position, *x*(*t*), is defined as the mean of the co-ordinates of the terminal particles, and is, therefore, independent of the configuration of the particles in the interior of the chain. Figure 4a shows the average chain velocity for a flexible chain at Γ=15 (*B*=0.43 mT) and a stiff chain at Γ=392 (*B*=2.2 mT). Both chains exhibit dynamic mode locking with steps at 
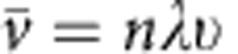
, as is also evident from the phase portraits for the first step (insets in Fig. 4a). However, the mode-locking steps for the flexible chain are remarkably wider than those for the stiff chain: the width of the first step increases by 16%, while for the second step this is almost 50%. Importantly, this points to a significant enhancement of the synchronization stability due to the increased flexibility of the chain.

The enhanced mode locking is clearly a collective dynamical effect that cannot be explained at the level of the average velocity. The simplest measure of the collective microscopic dynamics is the length of the chains, *l*, which is defined as the centre-to-centre distance between the terminal particles. Figure 4b,c compare the chain length and velocity for the stiff and flexible chains respectively. The stiff chain has a roughly constant length, and there is no correlation between the chain length and the instantaneous velocity. Strikingly, however, the length of the flexible chain fluctuates at the frequency of the chain velocity fluctuations, but exactly out of phase. This regular oscillation points to the presence of an internal breathing mode tied to the motion of the chain as a whole^50^. The contrast between the two chains is highlighted by the phase portraits for the chain length shown in Fig. 4d,e. For the stiff chain, this ‘length portrait' is stationary, indicative of the constant chain length. For the flexible chain, however, the phase portrait shows the closed loop form characteristic of a regular locked oscillation. This indicates that not only is the chain breathing, but that the breathing mode is in fact locked, leading to enhancement of the mode locking stability of the whole chain.

The fact that the velocity and length oscillations are exactly out of phase implies that the flexible chain decreases in length as it accelerates, and is shortest when travelling at its highest velocity. This suggests that chain motion proceeds by the back particle moving forward and compressing the chain, causing the particles in front of it to move. When the front particle is pushed, it moves to a new minimum on the landscape, relaxing and thus lengthening the chain. This process of ‘caterpillar' motion may be described as the periodic motion of a density wave or ‘kink' along the chain^50^. In this mobile chain, the density wave would move along the entire chain as the chain moves forward just one lattice spacing on the potential energy landscape. The speed of the density wave motion in such a short chain is, therefore, over five times faster than the motion of the chain as a whole, therefore resolving it in a chain of closely spaced Brownian particles is highly challenging. Furthermore, the close proximity of the particles makes it possible that the effect of the density wave is convoluted with hydrodynamic coupling^40^,^44^, though it is an interesting and open question as to how this affects the collective dynamics of the breathing chain and density wave.

### Kink dynamics

**Figure 5:**
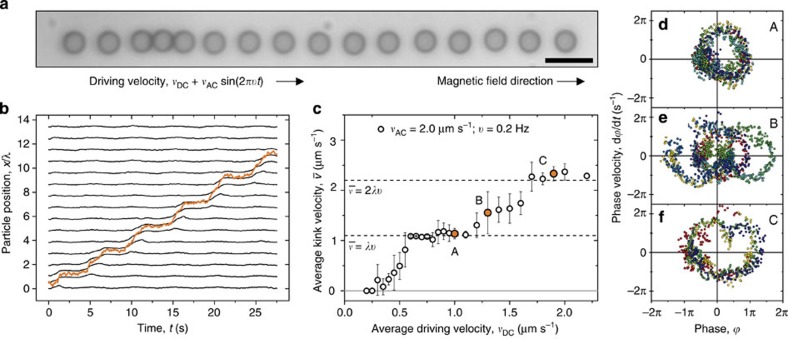
Dynamic mode locking of a kink. The kink is driven by a DC and AC drive in a stationary chain of 16 particles, pinned to 15 minima of a periodic optical potential energy landscape with a wavelength *λ*=5.5 μm. Particles are magnetically coupled, with Γ=20. (**a**) Microscopy image in which the kink is seen as a local density increase; scale bar, 10 μm. (**b**) Trajectories of all 16 particles in **a**, when driven by DC and AC drives, with *v*_DC_=2.0 μm s^−1^, *v*_AC_=2 μm s^−1^, and *ν*= 0.2 Hz. Orange line shows kink trajectory. (**c**) Average kink velocity 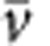 as a function of average driving velocity *v*_DC_, for AC drive with amplitude *v*_AC_=2 μm s^−1^ and frequency *ν*=0.2 Hz. All points are the result of four repeats, error bars show the standard deviation of repeats. (**d**–**f**) Phase portraits A–C correspond to the highlighted points in **c**.

To visualize the dynamics of such a density wave, we slow it down by both lengthening the chain and increasing the lattice spacing. We further simplify the situation by considering a static chain, as even the mobile chain studied above moves little on the timescale of density wave motion. We pin a chain of 16 particles in a strong potential energy landscape of 15 minima (trap spacing: *λ*=5.5 μm; laser power per trap: *W*≈1.75 mW; trap stiffness: *k*=8.7 × 10^−7^ kg s^−2^; trap depth: *V*_0_=200 *k*_B_*T*), as shown in Fig. 5a. The ‘extra' particle is forced to lie along the axis using a weak magnetic field (*B*=0.5 mT, Γ=20), thereby generating a kink. The position of this kink is determined from the reduced local number density 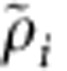, via a weighted average: 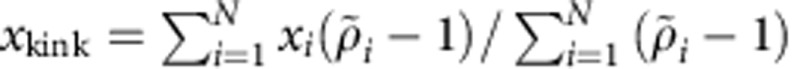. The reduced local number density is 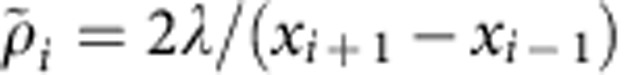, where *λ* is the wavelength of the sinusoidal optical potential energy landscape, and *x*_*i*_ is the position of particle *i*, such that far away from the kink, 
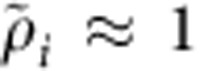
.

Figure 5b shows that when the kink is exposed to constant and modulated driving velocities, it is able to move along the whole chain, while the individual particles do not move more than one wavelength of the potential. Strikingly, by measuring the average kink velocity over a range of driving velocities, we show that the kink displays dynamic mode locking, as is evident from the plateaus in Fig. 5c. Moreover, going beyond the average velocity level, we present phase portraits corresponding to points A–C on the ‘staircase' in Fig. 5d–f. Points A (Fig. 5d) and C (Fig. 5f) show closed-loop phase portraits, confirming that the motion on the steps is periodic. By comparison with the phase portraits for single particles (see Fig. 2a), it is clear that these steps correspond to 1(1,0) and 2(2,0) modes. Conversely, the phase portrait for point B (Fig. 5e) shows a quasi-periodic trajectory, as this point is not on a mode-locked step. These three phase portraits are closely comparable to those for the equivalent points B–D in Fig. 2a. The microscopic dynamics of a driven kink in a coupled system are thus remarkably analogous to those of a single particle.

## Discussion

We describe the microscopic dynamics of synchronization in three driven colloidal model systems. All three display the characteristic synchronization plateaus in their ‘force–velocity' profiles^25^, demonstrating that colloidal particles in optical potential energy landscapes form a robust model for the study of dynamic mode locking. The tunability and manipulability of colloidal systems gives this model a broad scope: here, we move from single particles to chains and quasi-particle kinks, and there is huge potential to explore further. The three one-dimensional cases laid out here offer insight into the dynamics of technologically important but difficult to observe systems, including the motion of vortex lines^17^,^18^ and charge density waves^23^,^24^, indicating that future experiments in (for example) two-dimensional or aperiodic potential landscapes could prove fruitful. Moreover, the use of phase portraits to depict and differentiate dynamic modes allows analysis and comparison of the persistence and stability of locked states. Our analysis of mode locking in a driven colloidal chain, where a breathing mode apparently works to enhance the stability of a synchronized state, is only possible due to access to particle-level information. Furthermore, the ease with which experimental parameters may be changed has allowed us to design a density wave into a chain, making it possible to test the behaviour of this internal structure in simpler conditions. Our experiments unveil the microscopic dynamics that govern synchronization phenomena in driven systems, which can be challenging to capture in theory and simulation, especially in coupled driven systems where the role of hydrodynamic coupling remains an open question^40^,^44^,^45^. We believe that understanding and controlling these microscopic dynamics is crucial in achieving tunable synchronization behaviour in complex driven systems.

## Methods

### Colloidal model system

The colloidal particles used are Dynabeads M-270 carboxylic acid (diameter 3 μm), dispersed in 20% EtOH_aq_. These particles have a much higher density than the solvent, such that they sediment onto the wall of the quartz glass sample cell, forming a quasi-two-dimensional system. Iron oxide nanoparticles in the polystyrene matrix make the particles super-paramagnetic, such that they obtain a magnetic dipole moment when an external magnetic field is imposed, but have no magnetic moment in the absence of the external field. The strength of the dipolar inter-particle attraction is expressed as an interaction parameter Γ, which gives the interaction energy at contact of a pair of particles of diameter *σ* relative to *k*_B_*T*: 
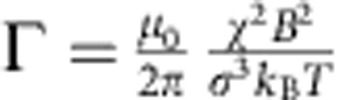
, where *μ*_0_ is the vacuum permeability, *χ*=6.7 × 10^−12^ A m^2^ T^−1^ is the magnetic susceptibility of the particles and *B* is the magnetic field strength.

### Optical tweezing and imaging

The optical tweezers setup is described in ref. 51, and, in short, consists of a 1064, nm laser, steered by a pair of perpendicular acousto-optical deflectors, and focused from above the sample using a Leica × 50, NA=0.55 long working distance microscope objective. We apply laser powers up to 1.75 mW per trap, which is well below the threshold when particle heating effects can come into play^56^. Imaging in the inverted configuration is done via a Zeiss × 40, NA=0.50 long working distance microscope objective, and a Ximea CMOS camera, protected by an IR bandpass filter. Magnetic fields are imposed using two permanent magnets, positioned equidistant from the sample.

### Image analysis

Time-stamped particle co-ordinates are obtained in real time at 40 Hz using template particle tracking, and particle trajectories, *x*(*t*), are numerically differentiated to find instantaneous velocity, *v*(*t*). Average particle velocity, 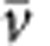, is determined by linearly fitting to an integer number of periods of the trajectory. Phase, *ϕ*, is found from particle position: 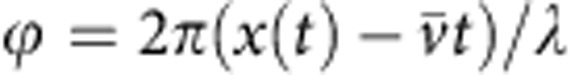, and phase velocity from particle velocity: 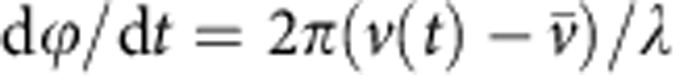. The chain position, *x*(*t*), is defined as the mean of the co-ordinates of the terminal particles, and chain length, *l*, is defined as the difference between the co-ordinates of the terminal particles. Phase is found from chain position as above. Kink position is determined from the reduced local number density 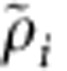, via a weighted average: 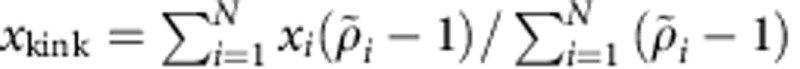. The reduced local number density is 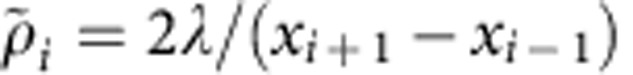, where *λ* is the wavelength of the sinusoidal optical potential energy landscape and *x*_*i*_ is the position of particle *i*, such that far away from the kink, 
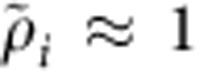
. The average kink velocity, 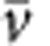, is determined by linearly fitting to an integer number of periods of the kink trajectory.

## Additional information

**How to cite this article:** Juniper, M. P. N. *et al*. Microscopic dynamics of synchronization in driven colloids. *Nat. Commun.* 6:7187 doi: 10.1038/ncomms8187 (2015).
